# Antibiotic prophylaxis in the surgical management of miscarriage in low-income countries: a cost-effectiveness analysis of the AIMS trial

**DOI:** 10.1016/S2214-109X(19)30336-5

**Published:** 2019-08-08

**Authors:** Ilias Goranitis, David M Lissauer, Arri Coomarasamy, Amie Wilson, Jane Daniels, Lee Middleton, Jonathan Bishop, Catherine A Hewitt, Andrew D Weeks, Chisale Mhango, Ronald Mataya, Iffat Ahmed, Olufemi T Oladapo, Javier Zamora, Tracy E Roberts

**Affiliations:** a Health Economics Unit, Institute of Applied Health Research, University of Birmingham, Birmingham, UK; b Institute of Metabolism and Systems Research, University of Birmingham, Birmingham, UK; c Clinical Trials Unit, Institute of Applied Health Research, University of Birmingham, Birmingham, UK; d School of Health Sciences, University of Nottingham, UK; e Institute of Translational Medicine, University of Liverpool, Liverpool, UK; f College of Medicine, Department of Obstetrics and Gynaecology, Blantyre, Malawi; g The Aga Khan University Hospital and Medical College Foundation, Karachi, Pakistan; h UNDP/UNFPA/UNICEF/WHO/World Bank Special Programme of Research, Development and Research Training in Human Reproduction (HRP), Department of Reproductive Health and Research, World Health Organization, Geneva, Switzerland; i Hospital Universitario Ramón y Cajal, CIBER en Epidemiología y Salud Pública (CIBERESP) and Instituto de Investigación Sanitaria (IRYCIS), Madrid, Spain

## Abstract

**Background:**

There is ongoing debate on the clinical benefits of antibiotic prophylaxis for reducing pelvic infection after miscarriage surgery. We aimed to study the cost-effectiveness of antibiotic prophylaxis in the surgical management of miscarriage in low-income countries.

**Methods:**

We did an incremental cost-effectiveness analysis using data from 3412 women recruited to the AIMS trial, a randomised, double-blind, placebo-controlled trial designed to evaluate the effectiveness of antibiotic prophylaxis in the surgical management of miscarriage in Malawi, Pakistan, Tanzania, and Uganda. Economic evaluation was done from a health-care-provider perspective on the basis of the outcome of cost per pelvic infection avoided within 2 weeks of surgery. Pelvic infection was broadly defined by the presence of clinical features or the clinically identified need to administer antibiotics. We used non-parametric bootstrapping and multilevel random effects models to estimate incremental mean costs and outcomes. Decision uncertainty was shown via cost-effectiveness acceptability frontiers. The AIMS trial is registered with the ISRCTN registry, number ISRCTN97143849.

**Findings:**

Between June 2, 2014, and April 26, 2017, 3412 women were assigned to receive either antibiotic prophylaxis (1705 [50%] of 3412) or placebo (1707 [50%] of 3412) in the AIMS trial. 158 (5%) of 3412 women developed pelvic infection within 2 weeks of surgery, of whom 68 (43%) were in the antibiotic prophylaxis group and 90 (57%) in the placebo group. There is 97–98% probability that antibiotic prophylaxis is a cost-effective intervention at expected thresholds of willingness-to-pay per additional pelvic infection avoided. In terms of post-surgery antibiotics, the antibiotic prophylaxis group was US$0·27 (95% CI −0·49 to −0·05) less expensive per woman than the placebo group. A secondary analysis, a sensitivity analysis, and all subgroup analyses supported these findings. Antibiotic prophylaxis, if implemented routinely before miscarriage surgery, could translate to an annual total cost saving of up to $1·4 million across the four participating countries and up to $8·5 million across the two regions of sub-Saharan Africa and south Asia.

**Interpretation:**

Antibiotic prophylaxis is more effective and less expensive than no antibiotic prophylaxis. Policy makers in various settings should be confident that antibiotic prophylaxis in miscarriage surgery is cost-effective.

**Funding:**

UK Medical Research Council, Wellcome Trust, and the UK Department for International Development.

## Introduction

Globally, about 210 million pregnancies are estimated to occur annually, 90% of which are in low-income countries.^1^ Almost 84 million (40%) of these pregnancies end in either miscarriage or induced abortion, in about equal proportions.^2^, ^3^ National and international guidelines on the surgical management of induced abortion advocate the use of antibiotic prophylaxis to reduce the risk of infection.^4^, ^5^, ^6^ These guidelines are underpinned by strong evidence on the clinical effectiveness of prophylactic antibiotics given before surgical abortion.^7^

By contrast, sufficient evidence to support the routine use of antibiotic prophylaxis in the surgical management of miscarriage is scarce,^8^ and current guidelines do not recommend the use of antibiotics before miscarriage surgery, unless there is evidence of infection.^9^ Surgical removal of miscarriage tissues is a most common method of miscarriage management,^10^ particularly in low-income countries where it represents up to 70% of all gynaecological admissions.^11^ Infection is a serious complication of miscarriage surgery and occurs in up to 30% of women in low-income countries,^12^ with potentially significant morbidity and mortality implications.^13^, ^14^ There is an urgent need for a more comprehensive evaluation of the potential role of antibiotic prophylaxis in this context,^8^ which has the potential to offer significant clinical and economic benefits to health systems globally.

In response to this, the Antibiotics In Miscarriage Surgery (AIMS) trial was jointly funded by the UK Medical Research Council, the Wellcome Trust, and the UK Department for International Development, as part of the Joint Global Health Trials Scheme, to evaluate the effectiveness of antibiotic prophylaxis during the surgical management of miscarriage in low-income countries. Here, we use data from the AIMS trial to determine the relative cost-effectiveness of antibiotic prophylaxis compared with placebo in the surgical management of miscarriage based on the outcome of cost per pelvic infection avoided within 2 weeks of surgery.

**Research in context**

**Evidence before the study**

In low-income countries, the surgical removal of miscarriage tissues is the most common method of miscarriage management. To date, national and international guidelines on the surgical management of miscarriage do not recommend the use of prophylactic antibiotics to reduce the risk of pelvic infection. Infections after miscarriage surgery are frequent in low-income settings and have been associated with clinically significant morbidity and mortality implications. A Cochrane review showed no previous high quality evidence of effectiveness, with four small single-centered studies showing no significant benefit from prophylactic antibiotics. The AIMS trial provided evidence on the role of antibiotic prophylaxis in miscarriage surgery.

**Added value of this study**

To our knowledge, this study is the first to assess the cost-effectiveness of antibiotic prophylaxis relative to placebo in women undergoing surgical management of miscarriage in low-income settings. We find that antibiotic prophylaxis in the surgical management of miscarriage is cost-effective and provides substantial economic and health benefits.

**Implications of all the available evidence**

For clinicians and policy makers there is now evidence of both clinical and cost-effectiveness. Our interpretation is that this new evidence should result in the recommendation that, in low-resource settings, antibiotic prophylaxis should be administered before miscarriage surgery. Guidelines on the management of miscarriage should be updated on the basis of this evidence.

## Methods

### Study design and participants

The AIMS trial was a multinational randomised, double-blind, placebo-controlled trial. Detailed information about the trial design and participants can be found in the published trial protocol^15^ and the clinical paper.^16^ Briefly, 3412 women with a spontaneous miscarriage (<22 weeks' gestation) undergoing surgical management of miscarriage with manual vacuum aspiration, suction curettage, or sharp curettage, and who were willing and able to provide informed consent, were recruited across 13 hospitals in Malawi, Pakistan, Tanzania, and Uganda between June, 2013, and April, 2017. Women were excluded from the study if they were younger than 16 years or had induced abortion of pregnancy, septic miscarriage or evidence of infection, allergy to prophylactic antibiotics (ie, doxycycline or metronidazole), used antibiotics within 7 days before randomisation, febrile illness, other contraindication to doxycycline and metronidazole, or a condition requiring immediate care, such as severe haemorrhage. Women were randomly assigned (1:1), using a secure internet facility, to receive a single preoperative dose (about 2 hours before surgery) of doxycycline (400 mg oral) and metronidazole (400 mg oral) or identical placebos. Although obesity is a key factor in the choice of dosage of prophylactic antibiotics in high-income countries, obesity was not an issue in the AIMS trial and, therefore, it was not formally considered. Women were asked to attend an in-person follow-up assessment on day 14 after treatment. No further formal assessment was made in participants who attended for this day 14 assessment. Those who did not attend were prompted by telephone (to themselves or their nominated contact person) to attend at a time convenient for them, or were offered in-person visits to their home address to minimise loss to follow-up. These additional attempts to contact those who did not attend for follow-up were permitted until day 28. Ethics approval was granted from the Liverpool School of Tropical Medicine Research Ethics Committee in the UK, the College of Medicine Research and Ethics Committee in Malawi, the National Council for Science and Technology in Uganda, the Ifakara Health Institute in Tanzania, and the Aga Khan University in Pakistan.

### Effectiveness outcome

The outcome of the AIMS trial was pelvic infections avoided within 2 weeks of surgery. As per trial protocol, pelvic infection was determined in the presence of two or more of the following clinical features: purulent vaginal discharge, pyrexia (>38°C), uterine tenderness on examination, and white cell count of more than 12 × 10^9^ cells per L, with no other recognised cause of infection, or only one of these four clinical features if there was a clinically identified need to administer antibiotics for the treatment of a presumed pelvic infection.

### Resource use and costs

Resource use information was collected prospectively via case report forms. Forms were completed on at least one occasion before discharge after surgery, at every contact assessment during the follow-up period, daily whilst an inpatient, and at final assessment. Resource use information was collected from the perspective of the health-care provider and included antibiotics and medications related to pain, allergy, diarrhoea, vomiting, nausea, malaria, and fever as well as inpatient hospital stays, outpatient visits, laboratory examinations, and treatment of complications for which antibiotic prophylaxis could potentially offer a clinical benefit. Such complications were haemorrhage requiring blood transfusion, repeat uterine evacuation, and anaphylaxis. Other rare complications, such as uterine perforations, were not considered as they do not relate to antibiotic prophylaxis and were expected to equally appear in both groups of the trial.

We calculated the cost associated with the use of antibiotics and other commonly prescribed medications on the basis of established dosage regimens and patient-specific duration of treatment. Mean unit costs were obtained from the International Drug Price Indicator Guide,^17^ which is recommended as the principal source for medication costs in low-income and middle-income settings.^18^ An adjustment of 25% was used to account for shipping, handling, and internal distribution costs (table 1).^18^, ^19^ Country-specific and hospital-specific unit cost estimates for secondary and tertiary patient services in each participating country were obtained from the WHO-CHOICE initiative.^20^ We used other secondary sources to estimate the cost of laboratory examinations and treatment of relevant complications.^21^, ^22^, ^23^, ^24^, ^25^ For women requiring blood transfusion, we used unit cost estimates for hospital-based blood transfusion services.^22^, ^23^ Appropriate cost estimates for repeat surgical removal of pregnancy tissues were used according to whether the surgery was done using dilatation and curettage or manual vacuum aspiration.^24^, ^25^ All non-medication-related unit costs used in the analysis are shown in table 2.

**Table 1:** Medication-related unit costs by severity (US$, 2016 price base)

		**Unit cost**
Antibiotic prophylaxis		$0·091
Pain		
	Mild	$0·049
	Moderate	$0·093
	Severe	$0·835
Allergy		
	Mild	$0·010
	Moderate	$0·103
	Severe	$1·614
Diarrhoea		
	Mild	$0·551
	Moderate	$3·849
	Severe	$5·361
Vomiting		
	Mild	$0·036
	Moderate	$0·641
	Severe	$5·239
Fever*		
	Mild	$0·049
	Moderate	$0·049
	Severe	$0·049
Nausea		
	Mild	$0·036
	Moderate	$0·855
	Severe	$1·768
Malaria		
	Mild	$0·434
	Moderate	$1·713
	Severe	$1·980
Infection		
	Mild	$0·063
	Moderate to severe	$1·693

Source: International Drug Price Indicator Guide (2015).^17^

* Main treatment is reflected on antibiotics use and investigations.

**Table 2:** Other resource use categories and associated unit costs (US$, 2016 price base)

		**Malawi**	**Pakistan**	**Tanzania**	**Uganda**	**References**
Hospital services*						
Inpatient stay						
	Per day in tertiary hospital	$4·41	$13·76	$8·38	$6·58	20
	Per day in secondary hospital	$3·23	$10·08	$6·13	$4·81	20
Outpatient visit						
	Per day in tertiary hospital	$1·22	$2·64	$2·45	$2·15	20
	Per day in secondary hospital	$0·83	$1·78	$1·65	$1·45	20
**Laboratory examinations**						
	White cell count	$1·10	$3·18	$2·10	$1·69	21
	Vaginal swab	$0·76	$2·19	$1·45	$1·17	21
	Blood culture	$2·73	$7·89	$5·21	$4·20	21
	Urinalysis	$1·00	$2·89	$1·91	$1·54	21
**Complications**						
Hospital-based blood transfusion (per unit of transfusion-ready blood)		$20·02	$58·45	$21·35	$31·03	22, 40
Repeat evacuation						
	Dilatation and curettage	$18·25	$46·60	$34·81	$28·07	24, 25
	Manual vacuum aspiration	$3·99	$18·05	$7·61	$6·14	24, 25

* These estimates only show the accommodation component of hospital costs; ie, excluding the cost of drugs and diagnostic tests but including personnel, capital, and food costs.

To standardise unit costs across countries in cases where data were not available, an index table was used to indicate the relative mean cost of tertiary and secondary inpatient and outpatient hospital services for each country-pair in the study, based on the WHO-CHOICE estimates (appendix p 1). This market-basket approach was used in the Disease Control Priorities Project that aimed to inform disease control priorities in low-income countries using economic evaluation,^18^ and it is an established costing method for the development of a complete set of country-specific unit cost data in multinational trials.^26^ All unit costs were adjusted to 2016 US$ using the average US inflation rate between the price base year used in individual studies and 2016, as recommended when there is a relatively high proportion of imported commodities in economic analyses.^27^ Given that the follow-up period was 2 weeks, costs were not discounted.

### Statistical analysis

Evidence suggests that health-care service provision and patient outcomes differ across different hospitals within one country and across countries,^28^ with these differences being particularly profound in low-income settings. Thus, owing to the multinational nature of the AIMS trial and the differences identified in costs and clinical outcomes across countries and hospitals, we used a multilevel random effects model to estimate the differences in mean costs and outcomes between the antibiotic prophylaxis and placebo trial groups. Multilevel modelling accounts for unobserved hospital-specific and country-specific effects on costs and outcomes and allows for the estimation of cost-effectiveness across the whole sample and for the individual participating countries.^29^

We did an incremental cost-effectiveness analysis to compare the costs and outcomes associated with the two groups of the trial. To account for the inherent uncertainty around cost-effectiveness point estimates, we used non-parametric bootstrapping with multilevel models to generate 1000 paired estimates of incremental mean costs and outcomes adjusted for age, marital status, gestational age, previous miscarriage or stillbirth, evidence of induced abortion, HIV status, type of miscarriage surgery, cadre of surgeon (ie, specialist doctor, non-specialist doctor, or non-physician, including midwives, nurses, and other clinical staff), and residential characteristics. We used the bootstrapping results to derive cost-effectiveness acceptability frontiers,^30^ which plot the probability of the optimal strategy being cost-effective across a range of values of willingness-to-pay per additional unit of outcome (ie, per pelvic infection avoided within 2 weeks of surgery).

The economic evaluation relied on available case analysis since only 74 (2%) of 3412 women were censored at 2 weeks follow-up. Given that group allocation was found to be a predictor of missingness in the AIMS trial data (ie, women in the antibiotic prophylaxis group were more likely to be missing),^16^ we did an additional multiple imputation analysis under the assumption that data were missing not at random. Although in this clinical context it was expected that censored women did not develop any pelvic infection, the analysis was done under the assumption that all censored women developed pelvic infection as a worst-case scenario. The multiple imputation was done using chained equations.^31^ Differences between the antibiotic prophylaxis group and placebo in terms of mean costs and outcomes from the 10 multiply imputed datasets were obtained according to Rubin's rules,^32^ using multilevel random effects models.^33^, ^34^

We did subgroup analyses to assess the relative cost-effectiveness of antibiotic prophylaxis for each participating country, for the different types of miscarriage (incomplete and missed miscarriage), for the different types of miscarriage surgery (manual vacuum aspiration, suction curettage, sharp curettage), and for different gestational age groups (<12 weeks and ≥12 weeks). To assess the robustness of the main study findings, we did a sensitivity analysis using published regional unit cost estimates for each participating country (appendix p 2).^18^ We used Stata (version 14.2MP) for all analyses.

### Role of the funding source

The funder of the study had no role in study design, data collection, data analysis, data interpretation, or writing of the report. The corresponding author had full access to all the data in the study and had final responsibility for the decision to submit for publication.

## Results

Between June 2, 2014, and April 26, 2017, 3412 women with a median age of 25 years (IQR 18–31) were recruited across Malawi (2145 [63%] of 3412), Pakistan (353 [10%]), Tanzania (210 [6%]), and Uganda (704 [21%]), and were assigned to receive either antibiotic prophylaxis (1705 [50%] of 3412) or placebo (1707 [50%] of 3412). Of the 3412 women recruited, 2876 (84%) were married, 2352 (69%) lived in an urban area, and 2086 (61%) were unemployed or in a housewife role. In terms of residential characteristics, 1060 (31%) women were living in rural areas, 589 (17%) had a flushing toilet, and 772 (23%) had non-shared piped and tapped water. 1262 (37%) of 3412 reported that they had problems getting the food they need sometimes or more often. 696 (21%) of 3412 women had previously had a miscarriage or stillbirth. Further information is available in the appendix (p 3).

158 (5%) of 3412 women developed pelvic infection within 2 weeks of surgery. Of these women, 68 (43%) were in the antibiotic prophylaxis group and 90 (57%) in the placebo group. The absolute risk difference between antibiotic prophylaxis and placebo per woman was −1·3% (95% CI −2·8 to 0·2; table 3). The strict definition of pelvic infection using the clinical criteria only (ie, excluding presumed infections in the presence of one clinical feature) resulted in a significant difference in the risk of pelvic infection between the two groups at the 5% level (risk ratio 0·60 [95% CI 0·37–0·96]; p=0·03).^16^ The clinical findings of the AIMS trial have been published in full elsewhere.^16^

**Table 3:** Mean per-woman costs (US$, 2016 price base) and risk of pelvic infection

		**Antibiotic prophylaxis (n=1660); raw mean (SD)**	**Placebo (n=1680); raw mean (SD)**	**Difference (antibiotic prophylaxis − placebo)**		
				Adjusted mean*	Normal-based 95% CIs†	Normal-based 95% CIs†
Mean per-woman costs (US$)						
	Pre-surgery prophylactic antibiotics	0·084 (0·000)	0·000 (0·000)	0·084	..	..
	Post-surgery antibiotics	0·797 (2·964)	1·068 (3·535)	−0·270	−0·490	−0·050
	Other medications	0·176 (1·603)	0·212 (2·593)	−0·031	−0·179	0·117
	Laboratory examinations	0·113 (0·499)	0·154 (0·730)	−0·045	−0·086	−0·005
	Complications	0·152 (1·678)	0·262 (3·274)	−0·121	−0·308	0·066
	Hospital services	0·280 (2·452)	0·385 (2·406)	−0·112	−0·287	0·063
	Total cost (country-specific unit cost estimates)	1·601 (6·419)	2·082 (8·564)	−0·496	−1·019	0·026
	Total cost (regional unit cost estimates)	2·350 (12·548)	3·040 (14·803)	−0·718	−1·693	0·257
Risk of pelvic infection		0·041 (0·198)	0·054 (0·225)	−0·013	−0·028	0·002

* Adjusted for age, marital status, gestational age, previous miscarriage or stillbirth, evidence of induced abortion, HIV status, type of miscarriage surgery, surgeon, and residential characteristics.

† CIs calculated via bias-corrected and accelerated bootstrapping (1000 replications).

The resource utilisation per group is shown in the appendix (p 4). Post-surgery antibiotics prescribed for pelvic or other infections, such as urinary tract infection and respiratory infection, were the biggest driver of costs, accounting for about 50% of the total cost in the two groups of the trial (table 3). In terms of post-surgery antibiotics, the antibiotic prophylaxis group was US$0·27 (95% CI 0·05–0·49) less expensive per woman relative to the placebo group. This finding is explained by the additional days of infection (about 180 days), and possibly more severe cases of infections, in the placebo group. Hospital-services-related costs were the second largest driver of costs, accounting for about 20% of the total costs. This finding is due to the 150 days of inpatient stay (53% of which were in the placebo group) and 164 outpatient visits (61% of which were in the placebo group) during the trial. The antibiotic prophylaxis group was $0·50 (95% CI 0·03–1·02) less expensive per woman than the placebo group was. In a sensitivity analysis, using the regional unit cost, estimates increased the mean per woman cost difference to $0·72 (95% CI 0·26–1·69).

The antibiotic prophylaxis group was more effective and less expensive than the placebo group was (dominant intervention). The figure shows the probability of antibiotic prophylaxis being cost-effective across a range of willingness-to-pay values per additional pelvic infection avoided in the main and secondary analyses. Antibiotic prophylaxis has a more than 80% probability of being cost-effective for any willingness-to-pay value below $20 per pelvic infection avoided (figure). According to WHO, for highly cost-effective interventions, decision makers should be willing to pay the country's per capita gross domestic product (GDP) for an additional disability-adjusted life-year (DALY) avoided.^35^ Other sources recommend that this willingness-to-pay in low-income settings should be a maximum of 50% of the per capita GDP.^36^ Thus, according to these sources, decision makers in the trial context should be willing to pay up to $224 (50% of the weighted per capita GDP) or $566 (weighted per capita GDP) for one DALY avoided (appendix p 5). Pelvic infections have a disability weight of 0·169,^37^ and are known to last up to 2 weeks, which results in 0·0065 DALYs. If decision makers are willing to pay $224 or $566 per DALY averted, this payment equates to $1·45 or $3·67 for 0·65% of a DALY. At these willingness-to-pay values per pelvic infection avoided, there is 97–98% probability that antibiotic prophylaxis is a cost-effective intervention (figure A). In the secondary, worst-case scenario analysis, where all censored women were assumed to have pelvic infection, antibiotic prophylaxis had 88–89% probability of being cost-effective (figure B). The downward slopes of the figure show that decision uncertainty is mainly driven by costs rather than outcomes. The conclusions drawn from the cost-effectiveness analysis were robust to the regional (sensitivity) analysis and all subgroup analyses, including the estimation of cost-effectiveness within each participating country (appendix p 6).

**Figure:**
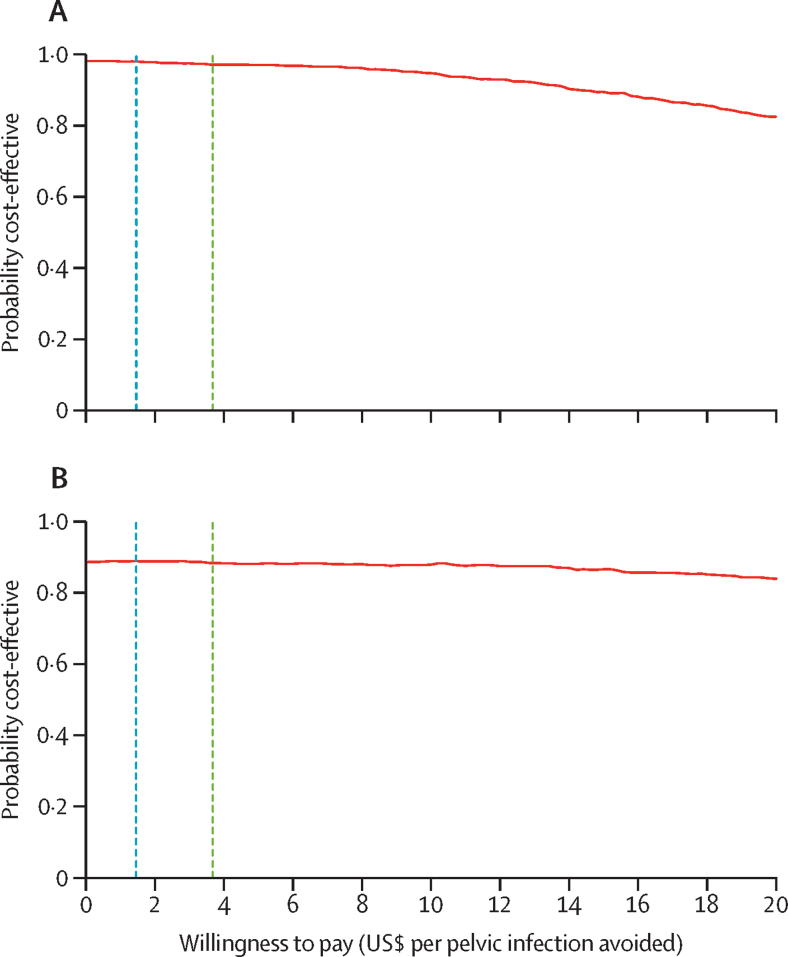
Cost-effectiveness acceptability frontier indicating the probability of antibiotic prophylaxis being cost-effective across different willingness-to-pay thresholds per pelvic infection avoided in the main (available case) analysis (A) and secondary (multiple imputation or worst-case scenario) analysis (B) The dashed lines show the expected decision maker's willingness-to-pay for a pelvic infection avoided, as estimated from Woods and colleagues^35^ (blue line) and the WHO recommendations (green line).^36^

## Discussion

This study assessed the cost-effectiveness of antibiotic prophylaxis relative to placebo in women undergoing surgical management of miscarriage in low-income settings. The findings show that the provision of antibiotic prophylaxis before miscarriage surgery results in fewer pelvic infections within 2 weeks of surgery and lower costs than the current practice of no antibiotic prophylaxis. Our analysis concluded that there is a 97–98% probability that antibiotic prophylaxis is a cost-effective intervention at expected thresholds of willingness-to-pay per additional pelvic infection avoided. This finding could translate to an annual total cost saving of up to $1·4 million if antibiotic prophylaxis was used routinely before miscarriage surgery across the four participating countries (Malawi, Pakistan, Tanzania, and Uganda) and a saving of up to $8·5 million across the two regions (sub-Saharan Africa and south Asia; appendix p 7).

Antibiotic resistance is a key global threat and reducing the unnecessary and inappropriate use of antibiotics is a global priority.^38^ However, we have shown that a single prophylactic dose of antibiotics before miscarriage surgery is an example of antibiotic usage that is both necessary and appropriate, and that it also reduced antibiotic usage after surgery.

To the best of our knowledge, this study is the first to assess the relative cost-effectiveness of antibiotic prophylaxis in the surgical management of miscarriage. The study benefited from a large sample size recruited from four low-income countries and 13 hospitals, a wide range of primary data, and a high follow-up rate. However, the study has some limitations. For pragmatic financial, time, and trial burden-related reasons, extensive bottom-up costing of all resource items was not done during the trial. Although this decision probably increased the uncertainty around the unit cost estimates used in the analysis, all estimates were obtained from valid and reputable sources, including the International Drug Price Indicator Guide, WHO, and other secondary sources based on thorough bottom-up costing. A sensitivity analysis using regional cost estimates led to similar results, which supports the generalisability of our findings.

In low-income settings, managing miscarriage complications can involve substantial out-of-pocket expenses,^39^ such as travel expenses, formal or informal expenses for outpatient services and inpatient care, and time off work for women and their family members. Given the emotionally distressing period after pregnancy loss, these expenses were not captured in the trial to avoid adding further burden to participants. Nevertheless, the increased complication rates seen in the placebo group suggest that these costs—in a societal perspective of analysis—probably led to an even larger cost difference between the two groups. Pelvic infections can also result in longer-term effects, such as pelvic pain, ectopic pregnancy, and infertility. Cost and disability effects associated with these outcomes were not considered in the analysis. However, given that pelvic infections were more frequent in the placebo group, an exploration of these costs and outcomes in a lifetime modelled time-horizon would improve the cost-effectiveness of antibiotic prophylaxis even further.

Finally, although only 74 (2%) of 3412 women were censored at 2 weeks after miscarriage surgery, women in the antibiotic prophylaxis group were more likely to be missing. This finding could be attributed to the small number of women lost to follow-up, or it might indicate better effectiveness for antibiotic prophylaxis, but a worst-case scenario was used for analysis. For this calculation, costs for censored women were multiply imputed assuming the presence of pelvic infection. This analysis concluded that, at expected thresholds of willingness-to-pay per additional pelvic infection avoided, there is 88–89% probability that antibiotic prophylaxis is cost-effective.

Current international guidelines recommend the use of prophylactic antibiotics in abortion surgery but not in miscarriage surgery. Our findings lend strong support on the cost-effectiveness of antibiotic prophylaxis before miscarriage surgery. Policy makers in various settings should be confident that providing prophylactic antibiotics in the surgical management of miscarriage is a good use of their restricted health-care budgets. Decision making guidelines on miscarriage management should be updated to reflect the findings of this study and the efficacy evidence from the AIMS trial.

## Data sharing

Any data requests for the clinical data should be made to the corresponding author of the AIMS clinical paper.^16^ No additional data are available for this cost analysis.
