# Supplementary material for: Antibiotic prophylaxis in the surgical management of miscarriage in low-income countries: a cost-effectiveness analysis of the AIMS trial
Source: Lancet Glob Health. 2019 Aug 8;7(9):e1280–6. doi: 10.1016/S2214-109X(19)30336-5 (PMC6695526; doi:10.1016/S2214-109X(19)30336-5)
Supplement: Supplementary appendix [file mmc1.pdf]

THE LANCET
Global Health

Supplementary appendix

This appendix formed part of the original submission and has been peer reviewed. We post it as supplied by the authors.

Supplement to: Goranitis I, Lissauer DM, Coomarasamy A, et al. Antibiotic prophylaxis in the surgical management of miscarriage in low-income countries: a cost-effectiveness analysis of the AIMS trial. *Lancet Glob Health* 2019; **7:** e1280–86.

TABLE OF CONTENTS

Appendix 1: Relative hospital cost indices of participating countries 1

Appendix 2. Unit costs used in the sensitivity/ regional analysis (US$, 2016 price base) 1

Appendix 3: Sample characteristics 2

Appendix 4: Number of women per resource use item.. 3

Appendix 5: Willingness-to-pay (US$) estimates for a disability-adjusted life-year and pelvic infection avoided in the four participating countries 4

Appendix 6: Mean per-woman cost and risk of pelvic infection across participating countries (US$, 2016 price base) 5

Appendix 7: Budget impact analysis 6

References 7

Appendix 1: Relative hospital cost indices of participating countries

**To country**	**From country**			
	**Malawi**	**Uganda**	**Tanzania**	**Pakistan**
Malawi	1·00	0·65	0·52	0·34
Uganda	1·55	1·00	0·81	0·53
Tanzania	1·92	1·24	1·00	0·66
Pakistan	2·92	1·88	1·52	1·00

Appendix 2. Unit costs used in the sensitivity/ regional analysis (US$, 2016 price base)

**Resource use items**	**Sub-Saharan African countries**	**Pakistan**
Inpatient stay (per day)	15·08	15·93
Outpatient visit (per day)	4·29	4·83
Cost per unit of safe blood transfused	22·54	23·57
Surgery	46·12	72·45

Source: The Disease Control Priorities Project (DCPP)—Working paper No. 9 (2005) [1]

Appendix 3: Sample characteristics

Sample characteristics (n = 3,412)	Frequency	%
Personal characteristics		
Age [median (25th –75th percentile): 25 (18-31)]		
Country		
Malawi	2,145	63
Uganda	704	21
Tanzania	210	6
Pakistan	353	10
Marital status		
Single	483	14
Married	2,876	84
Other	53	2
Educational status		
No education	1,044	31
Primary education	1,207	35
Secondary or higher	1,161	34
Employment status		
Employed	1,075	32
Unemployed	266	8
Housewife	1,820	53
Student	251	7
HIV positive		
No	3,346	98
Yes	66	2
Residential characteristics		
Area of residence		
Rural	1,060	31
Urban	2,352	69
Residence floor-type		
Cement/Stone/Tile	2,372	70
Other (e.g. mud, brick etc.)	1,040	30
Residence latrine-type		
No latrine	27	1
Non-ventilated pit latrine	2,437	71
Ventilated improved pit latrine (VIP)	359	11
Flush toilet	589	17
Residence water source		
Non-shared piped and tapped water	772	23
Shared piped and tapped water	2,521	74
Closed (e.g. river, lake) or open water	119	3
Lacking food		
Never	2,150	63
Sometimes	1,217	36
Often	45	1

Appendix 3: Sample characteristics (Continued)

Sample characteristics (n = 3,412)	Frequency	%
Pregnancy-related characteristics		
Planned pregnancy		
No	1,166	34
Yes	2,246	66
Diagnosis		
Missed miscarriage	552	16
Incomplete miscarriage	2,860	84
Gestation		
Less than 12 weeks	1,673	49
12 weeks or more	1,737	51
Previous miscarriage		
No	2,806	82
Yes	606	18
Previous stillbirth		
No	3,322	97
Yes	90	3
Procedure-related characteristics		
Type of miscarriage surgery		
Manual vacuum aspiration	791	23
Suction curettage	212	6
Sharp curettage	2,390	70
Cadre of surgeon		
Specialist doctor	439	13
Non-specialist doctor	2,591	76
Non-physician (i.e. midwife, nurse or clinician)	363	11

Appendix 4: Number of women per resource use item

**Resource use items**	**Antibiotic prophylaxis**		**Placebo**		*P* - value
	**Frequency**	**%**	**Frequency**	**%**	
Post-surgery antibiotics	130	7·82	163	9·70	0·06
Other medications	253	15·23	287	17·08	0·15
Laboratory examinations	108	6·50	128	7·62	0·21
Blood transfusion	1	0·06	4	0·24	0·18
Repeat evacuation	13	0·78	16	0·95	0·60
Hospital services	74	4·46	131	7·80	0·00
Outpatient services	56	3·37	108	6·43	0·00
Inpatient services	22	1·33	31	1·85	0·22

Appendix 5: Willingness-to-pay (US$) estimates for a disability-adjusted life-year and pelvic infection avoided in the four participating countries

**Recommendations**	**Outcome**	**Malawi**	**Uganda**	**Tanzania**	**Pakistan**	**Weighted mean**
WHO─per capita GDP[2]	DALY averted	355	719	950	1317	566
Woods et al. (2016)[3]	DALY averted	116	293	357	669	224
Estimate based on WHO recommendation	Pelvic infection averted	2·30	4·66	6·15	8·53	3·67
Estimate based on Woods et al. (2016) recommendation	Pelvic infection averted	0·75	1·90	2·31	4·33	1·45

Appendix 6: Mean per-woman cost and risk of pelvic infection across participating countries (US$, 2016 price base)

**Country**	**Antibiotic prophylaxis**	**Placebo**	**Difference (Antibiotic prophylaxis minus Placebo)**		
	**Raw mean (SD)**	**Raw mean (SD)**	**Adjusted mean§**	**95% CIs**	
Malawi (n = 2,145)					
Cost	1·901 (6·980)	2·236 (9·418)	**-0·408**	-1·129	0·313
Risk of pelvic infection	0·058 (0·234)	0·070 (0·255)	**-0·013**	-0·034	0·008
Uganda (n = 704)					
Cost	1·606 (6·935)	2·075 (7·886)	**-0·520**	-1·652	0·612
Risk of pelvic infection	0·018 (0·131)	0·028 (0·165)	**-0·013**	-0·035	0·009
Tanzania (n = 210)					
Cost	0·463 (1·668)	1·350 (5·775)	**-0·993**	-2·117	0·131
Risk of pelvic infection	0·009 (0·096)	0·021 (0·143)	**-0·014**	-0·047	0·019
Pakistan (n = 353)					
Cost	0·411 (1·584)	1·549 (4·927)	**-0·936**	-1·708	-0·162
Risk of pelvic infection	0·000 (0·000)	0·024 (0·152)	**-0·020**	-0·043	0·003

Appendix 7: Budget impact analysis

Estimates	Malawi	Uganda	Tanzania	Pakistan	All AIMS Trial countries	Sub-Saharan Africa and South Asia
Annual number of livebirths§	665,371	1,665,238	2,064,371	5,451,092	9,846,072	73,193,524
Annual number of miscarriagesǂ	146,382	366,352	454,162	1,199,240	2,166,136	16,102,575
Potential number of miscarriage surgeries (50% of miscarriages)	73,191	183,176	227,081	599,620	1,083,068	8,051,288
Potential number of miscarriage surgeries (75% of miscarriages)	109,786	274,764	340,621	899,430	1,624,602	12,076,931
Budget impact (assuming 50% of miscarriages are surgically managed)	$29,862	$95,252	$225,491	$561,244	$911,849	$5,635,901
Budget impact (assuming 75% of miscarriages are surgically managed)	$44,793	$142,877	$338,237	$841,867	$1,367,774	$8,453,852

§ Source: UNICEF (2016)[4]

ǂ Assuming the number of miscarriages is 20% of live births and 10% of all induced abortions[5]

References

1. Mulligan J, Fox-Rushby JA, Adam T, Johns B, Mills A. Unit costs of health care inputs in low and middle income regions. *Disease Control Priorities Project Working Paper* 2005; **9**.
2. World Health Organization (WHO). The world health report 2002: reducing risks, promoting healthy life: WHO; 2002.
3. Woods B, Revill P, Sculpher M, Claxton K. Country-level cost-effectiveness thresholds: initial estimates and the need for further research. *Value in Health* 2016; **19**(8): 929-35.
4. UNICEF. The State of the World’s Children 2016 Statistical Tables, 2016.
5. Singh S. Abortion worldwide: a decade of uneven progress: Guttmacher Institute, 2009.
